# Laterals take it better – Emerging and young lateral roots survive lethal salinity longer than the primary root in Arabidopsis

**DOI:** 10.1038/s41598-020-60163-7

**Published:** 2020-02-24

**Authors:** Vivek Ambastha, Yael Friedmann, Yehoram Leshem

**Affiliations:** 1 Department of Plant Sciences, MIGAL - Galilee Technology Center, 2 Tarshish St., POB 831, Kiryat-Shmona, 11016 Israel; 2 Bio-Imaging Unit, The Alexander Silberman Institute of Life Sciences, The Hebrew University of Jerusalem, Jerusalem, Israel

**Keywords:** Abiotic, Salt

## Abstract

Plant responses to salinity have been extensively studied over the last decades. Despite the vast accumulated knowledge, the ways Arabidopsis lateral roots (LR) cope with lethal salinity has not been fully resolved. Here we compared the primary root (PR) and the LR responses during events leading to lethal salinity (NaCl 200 mM) in Arabidopsis. We found that the PR and young LR responded differently to lethal salinity: While the PR died, emerging and young LR’s remained strikingly viable. Moreover, “age acquired salt tolerance” (AAST) was observed in the PR. During the 2 days after germination (DAG) the PR was highly sensitive, but at 8 DAG there was a significant increase in the PR cell survival. Nevertheless, the young LR exhibited an opposite pattern and completely lost its salinity tolerance, as it elongated beyond 400 µm. Examination of several cell death signatures investigated in the young LR showed no signs of an active programmed cell death (PCD) during lethal salinity. However, Autophagic PCD (A-PCD) but not apoptosis-like PCD (AL-PCD) was found to be activated in the PR during the high salinity conditions. We further found that salinity induced NADPH oxidase activated ROS, which were more highly distributed in the young LR compared to the PR, is required for the improved viability of the LR during lethal salinity conditions. Our data demonstrated a position-dependent resistance of Arabidopsis young LR to high salinity. This response can lead to identification of novel salt stress coping mechanisms needed by agriculture during the soil salinization challenge.

## Introduction

The ongoing process of soil salinization negatively affects many plant species, including staple crops, imposing a major threat for global food production^1,2^. Therefore, it is not surprising that over the last decades numerous studies have been dedicated to the understanding of plant responses to salt stress. These studies include those conducted from the organismic (whole plant) level to the cellular and molecular levels, deciphering both osmotic and ionic nature of salt^3–7^.

Since the root is the primary tissue that directly interacts with the rhizosphere’s saline environment, special attention has been given to root tissue. In many of the past but also present studies, salt stress responses were determined in whole root samples without differentiating between the root’s various developmental zones and cell types, mainly while being compared to the shoot^8,9^. Nevertheless, it is well established by now that each of the root’s different developmental zones responds to salt in a different manner. For example, the canonical Sodium/Proton antiporter NHX1 was found in Arabidopsis roots to be expressed during salt stress in the elongation zone but excluded from the root tip meristem, whereas SOS1 exhibited the opposite expression pattern^10^. Moreover, in a milestone study Dinneny *et al*. isolated several types of Arabidopsis root cells and showed that their transcriptomes differed significantly during salt stress^11^. Therefore, it is necessary to specify the exact cell’s identity and position along the root during that stress. It is important to note, though, that the salt concentration used in the above study was 140 mM NaCl, which is non-lethal^11^, as opposed concentrations of 200 mM NaCl or higher which were reported to be lethal^12,13^. Moreover, the above study focused on cells from the tip zone of the primary root (PR) and did not include cells from the branching lateral roots (LR), whose salt induced transcriptome has not yet been determined.

The LRs branch off the PR above the root hair zone, initiating from the pericycle layer, forming of a new meristem, which after differentiation resembles the PR meristem, eventually giving rise to a new functional root^14,15^. LR formation and development is known to be tightly regulated by Auxin^16^ as well as other phytohormones^17,18^. Interestingly, NADPH oxidase induced ROS, which are key secondary messengers in stress responses and stomatal ABA signaling^19^, were reported recently to facilitate LR emergence and development^20^.

LR formation and development is also affected by several environmental factors such as phosphate and nitrogen depletions in the soil^21,22^, and salinity which inhibited Arabidopsis LR as well as PR^23–25^. Furthermore, it has been reported that Arabidopsis PR and LR responded differently to mild salt stress where the salinity growth arrest was released in the PR much earlier than the LR, that remained inhibited for several more days^26^. It was further shown that this inhibition was recovered in the ABA insensitive1-1 mutant and that specific endodermal ABA signaling regulates the salt induced post emergence LR growth inhibition. Thus, root architecture was regulated in a way that is thought by these authors to “prevent root growth into saline environments”. Indeed, it is thought that PR and LR differential growth dynamics are essential for plant adaptation to various environmental conditions^27^.

Cell death processes such as apoptosis-like programmed cell death (AL-PCD) and Autophagic PCD (A-PCD) were studied in roots of several species during salt stress^28–30^. However, these studies focused mainly on the PR, while events leading to salinity induced cell death in Arabidopsis LR are still obscure. Moreover, to the best of our knowledge, salinity induced ROS which was well documented in Arabidopsis PR^12,13,31,32^, has not been previously studied in Arabidopsis LR during high-lethal salt treatments.

Prolonged exposure to high, lethal NaCl concentration (200 mM) was shown to completely inhibit Arabidopsis growth. This growth arrest can be attributed to cell death that was reported to occur in Arabidopsis roots during such harsh treatment^13^. However, despite commonly held views that at *lethal* salt concentrations (200 mM) both PR and LR cells do not survive, we reveal that Arabidopsis emerging and young LRs tolerated lethal salt concentrations much better and survived longer than the PR. We also discuss several survival pathways which are potentially involved.

## Results

### Viability assays in PR and LR during lethal salt treatments

**Figure 1:**
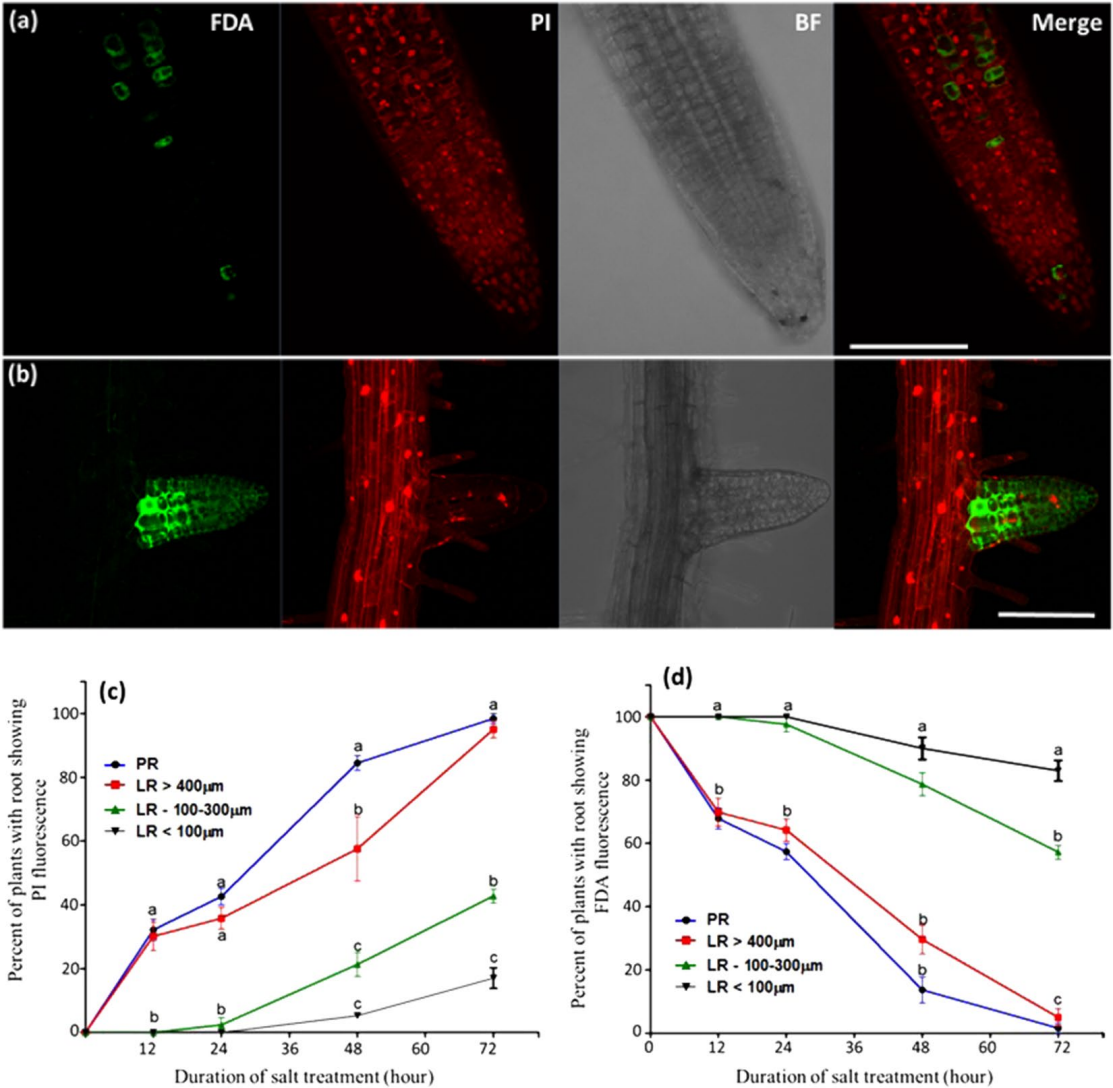
Viability of the PR and the LR during lethal salinity. Seven day old WT Arabidopsis seedlings were subjected to salt stress (200 mM NaCl + 1/2 MS) for 12, 24, 48 and 72 h and then stained with FDA + PI. Shown are representative confocal images of: (**a** and **b**) WT plants stained with FDA + PI in the PR and LR positions. The presented confocal images were captured after 48 hours of stress. Scale bars = 50 µm. (**c** and **d**) Graphical display of the confocal images presented in (**a**) and (**b**) during 0, 12, 24 and 72 hours of salt stress. The colored lines in the graphs indicate LR lengths (in µm). The experiments were repeated three times (n = 20 plants in each time point in all the experiment ± S.E.). Statistical analysis was done by Tukey’s Honest Significant Difference test (at P < 0.05) for each time point separately. (Significant differences are indicated by small letters). The shown images are projection of the entire z-stack at maximum intensity. The complete Z stack of the individual of the presented images can be found in Figs. SI1, SI2. Confocal images of non-stressed plants can be found in Fig. SI3.

To study the effect of lethal NaCl concentration (200 mM) on the cell viability in the different developmental zones of the root, the vital stains FDA and PI, were applied to 7 day old seedlings. Confocal microscopy examination of the fluorescent signals revealed that the emerging and young LR (shorter than 100 µm, mainly composed of dividing meristematic cells) exhibited profound salt tolerance as compared with the PR and elongated LR (longer than 400 µm, which included active meristem, elongation and mature zones): while the PR cells’ vitality dropped sharply 24–48 hours after stress (HAS), in all of its different developmental zones (meristem, elongation and mature zones), the cells in the young LR survived longer and remained highly viable even at 72 HAS (Fig. 1a–d. Presenting the PR’s division, elongation and mature zones. For the complete Z stacks of Fig. 1a,b see Figs. SI1 and SI2. Confocal images of non-stressed plants are presented in Fig. SI3).

Interestingly, the LR which were longer than 400 µm exhibited salt sensitivity exactly as the PR and were completely FDA negative from their tip up to the LR-PR junction. Nevertheless, LR which were 100–300 µm long, exhibited improved tolerance as compared with the PR but lower tolerance than the young LR (shorter than 100 µm) (Fig. 1).

**Figure 2:**
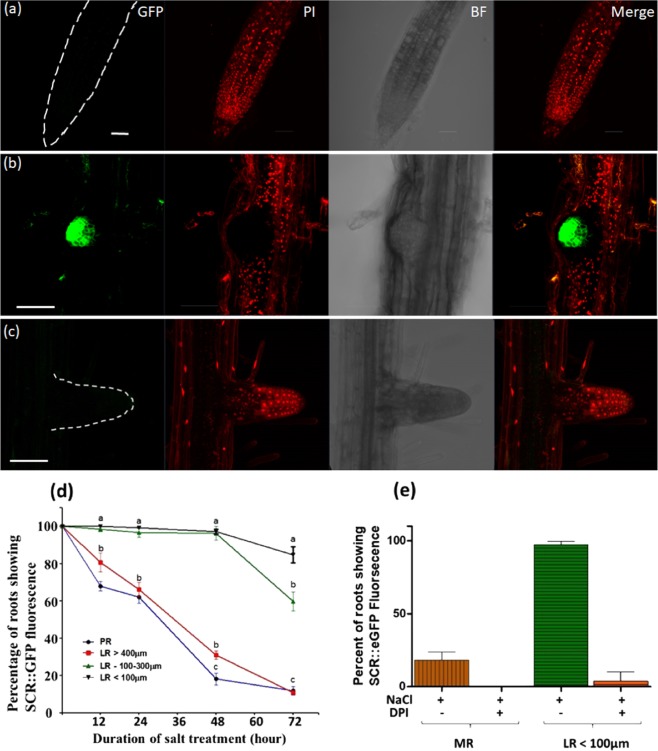
SCR::GFP fluorescence in the PR and the LR during lethal salinity. Seven day old SCR::GFP Arabidopsis seedlings were subjected to salt stress (200 mM NaCl + 1/2 MS) for 12, 24, 48 and 72 h and then stained with PI as in Fig. 1. Shown are representative confocal images of: (**a**,**b**) SCR::GFP plants during salt stress with (**c**) or without DPI. The confocal images were captured after 48 hours of stress. The GFP and PI fluorescent signals were captured as mentioned in methods. Scale bars = 50 µm. (**d**) Is graphical display of the GFP signal in (**a**–**c**) during 0,12, 24, 48 and 72 hours of salt stress. (**e**) Histogram of SCR::GFP fluorescence of the DPI treatment during 48 hours of salt stress. The experiments were repeated three times (n = 20 plants in each time point in all the experiment ± S.E.). Statistical analysis was done by Tukey’s Honest Significant Difference test (at P < 0.05) for each time point separately. (Significant differences are indicated by small letters). The shown images are projection of the entire z-stack at maximum intensity. The complete Z stack of the individual of the presented images can be found in Figs. SI4–SI6. Confocal images of non-stressed plants can be found in Fig. SI7.

PI staining was also performed during the same salt conditions in the following transgenic lines which express GFP specifically in different cell types of the root tip: SCR::GFP, WOL::GFP, COR::GFP and WOX5::GFP lines which mark the Endodermis and Quiescent Center (QC), stele and Cortex, respectively. Even after 72 HAS, normal and bright GFP signal was observed in the young LR cells, but no GFP signal was seen in the PR cells of these lines. Furthermore, the PI staining in the PR zone which lacked the GFP signal was localized in the nuclei, indicating death of cells in that region. On the other hand, PI nucleus staining was not observed at the LR zone that kept strong GFP expression, indicating therefore cell viability in that position (Fig. 2a,b,d, images presented only for the SCR::GFP line. The complete Z stack images of Fig. 2a–c are provided in Figs. SI4–SI6, respectively). In non-stressed SCR::GFP plants, strong and normal GFP signal was observed at the PR and LR, but no PI nuclei staining was seen in those root positions (Fig. SI7). To ensure that during the described experiments the LRs indeed experienced salt stress, plasmolysis was confirmed in meristematic as well as elongated cells of that position, by visualizing clear retraction of the protoplast from the cell-wall (Fig. SI8).

**Figure 3:**
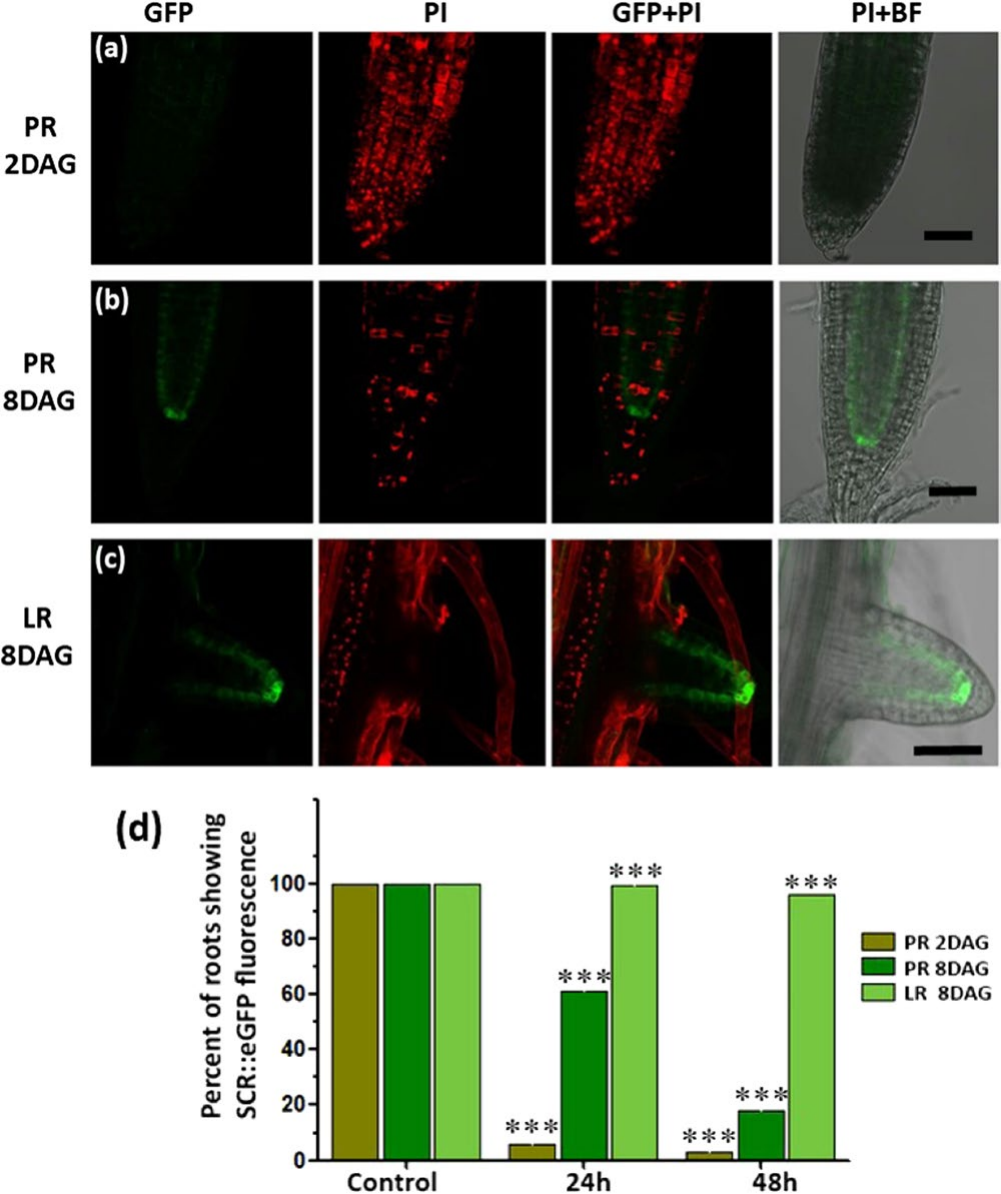
Age dependent salt sensitivity of the PR. 2 days after germination (DAG) PR is more sensitive to salt stress compare to LR of 8DAG. The SCR::GFP Arabidopsis plants of 2 and 8 DAG were subjected to lethal salt stress. Shown are the representative confocal images of PR of (**a**) 2 DAG plants that were salt treated for 24 h with no SCR::GFP fluorescence but ample of PI labelled nuclei indicating complete death of PR. (**b**) PR of 8 DAG SCR::GFP plants that were salt treated for 24 h, with SCR specific endodermal GFP expression and few PI labelled nuclei indicating induction of cell death processes. (**c**) Young LR (shorter than 100 µm) of 8 DAG SCR plants that were salt treated for 24 h, with clear SCR specific endodermal GFP expression, without any PI labelled nuclei. Note in (**c**) the presence of PI labelled nuclei at the PR site of LR emergence, indicating death of the PR cells at that position and the viability of the complete LR which was PI negative. (**d**) Quantification of percent of roots showing SCR::GFP fluorescence in PR or LR after 24 h and 48 h of lethal salt stress. Asterisk (***) above the bar in graph indicate statistical significance determined by pairwise Student’s t-tests (α = 0.05). The scale bars = 50 µm. The presented fluorescent images were projected at maximum intensity. The complete Z-stack images are provided in Figs. SI8–10.

Since dividing cells in the young LR meristems (shorter than 100 µm) were more tolerant to salt than the fully differentiated cells in the higher regions of the LR (longer than 400 µm), we wanted to further study the potential role of the meristem’s age and developmental stage on lethal salinity tolerance. Therefore, viability assay was performed (as above) in 2 and 8 days after-germination (DAG) SCR::GFP plants, which were subjected to lethal salinity for 24 or 48 hours. The PR of the 2 DAG plants which were stressed for 24 hours, exhibited 94% death (no SCR GFP fluorescence) (Fig. 3a,d). At the same time, the death rates of the PR from the 8 DAG plants were 38.9% only (Fig. 3b,d. Image of 3b, represents a survived PR), while the 8 DAG young LR showed no death at all (Fig. 3c,d). It should be pointed out that in the stressed 8 DAG PR, few PI labelled nuclei were observed in the PR periphery - external to the endodermis (Fig. 3b and Fig. SI9) indicating that induction of cell death processes has been initiated. During 48 HAS, the death rate of the PR from the 8 DAG plants increased to 78.7%, which was still lower than the 97% death rate observed in the 2 DAG PR (Fig. 3d). At that time the death rate of young LR from the 8 DAG plants was 4% only (Fig. 3d). Therefore, demonstrating again the increased salt resistance of young LR, that was presented above in Figs. 1 and 2. To find out whether the young LR actually experienced salt stress, we validated that clear retraction of the protoplast from the cell wall was observed there and that all LR cells exhibited plasmolysis (Fig. SI11).

### Cell death processes in the PR and LR during lethal salinity

**Figure 4:**
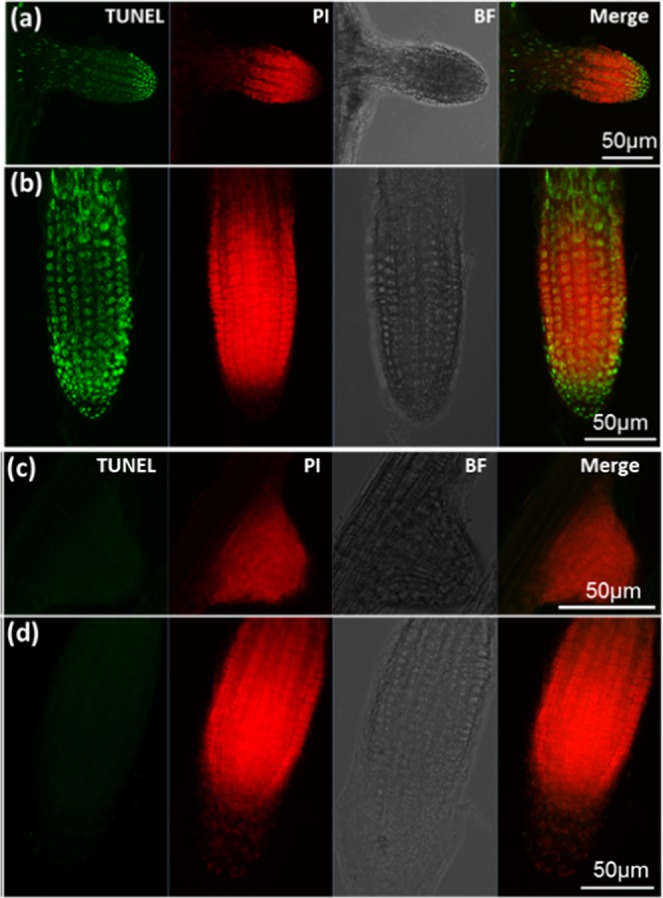
TUNEL labeling in PR and LR during lethal salinity. Roots of Arabidopsis plants that were treated with 200 mM NaCl for 24 or 48 hours, were labeled with TUNEL as mentioned in methods. (**a**,**b**) Shown are the representative confocal microscopy images of TUNEL positive control of LR and PR that were treated with DNase-I for 20 min. The green punctate structure represents nuclei with fluorescein labelled nick and PI was used as a counterstain to locate cell nuclei. (**c,d**) Are the representative images of TUNEL labeled roots that were salt stressed for 48 hours (similar results were obtained during 24 HAS). The lack fluorescein labelling in the LR (**c**) and PR (**d**) clearly indicate the absence of DNA nicking in these tissue under toxic salinity conditions. The experiment was repeated twice (n = 7–10 plants).

In order to characterize the exact type of cell death that might be taking place in Arabidopsis roots (of 8DAG plants) during lethal salinity we performed TUNEL staining – which indicates DNA single strand nicks and is one of the major hallmarks of apoptosis-like programmed cell death (AL-PCD)^33^. As expected, during 24–48 HAS no TUNEL positive nuclei were observed in young LR cells (Fig. 4c), which were shown earlier to be during this stress duration mostly viable (~100%, Figs. 1c and 2d). Nevertheless, no TUNEL positive nuclei were seen in the PR either (Fig. 4d). Since the TUNEL positive control successfully labeled both LR and PR nuclei (Fig. 4a,b), we can conclude that no DNA nicking has occurred in the stressed PR cells, which during 24–48 HAS reached ~40–80% mortality (Figs. 1c and 2d), and therefore we concluded that AL-PCD did not take place in these cells.

**Figure 5:**
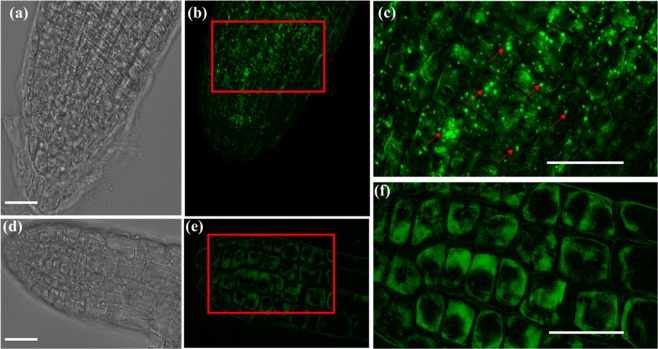
Salt stress Monodansylcadaverine (MDC)-labeled endosomes are induced in the primary root. Seven day old Arabidopsis seedlings were subjected to 200 mM NaCl for 24 hours. Roots were stained with MDC then analyzed by EVOS M5000 fluorescence microscopy using DAPI-specific LED cube. (**b**) MDC stained endosomes were observed in the PR (**e**), but not in the LR which lacked any bright punctate structures (**f**). (**a**) and (**d**) are the bright field image of PR and LR. (**c**) is the enlarged section of PR. In (**e**), the presence of MDC punctate structures are marked by red arrows. (**f**) is the enlarged image of LR, which lacked any bright punctate structures. Scale = 20 µm. Fluorescent images of MDC staining in the PR and LR of non-stressed plants is provided in Fig. SI13.

Since autophagy can lead to PCD in plants subjected to various stresses^34–38^ we treated the salt stressed plants with Monodansylcadaverine (MDC) which labels autophagic endosomes^39^. Fluorescent microscopy examination of these plants at 24 HAS revealed high numbers of autophagosomes in the PR cells (Fig. 5a–c), but no autophagosomes were detected in the LR (Fig. 5d–f).

**Figure 6:**
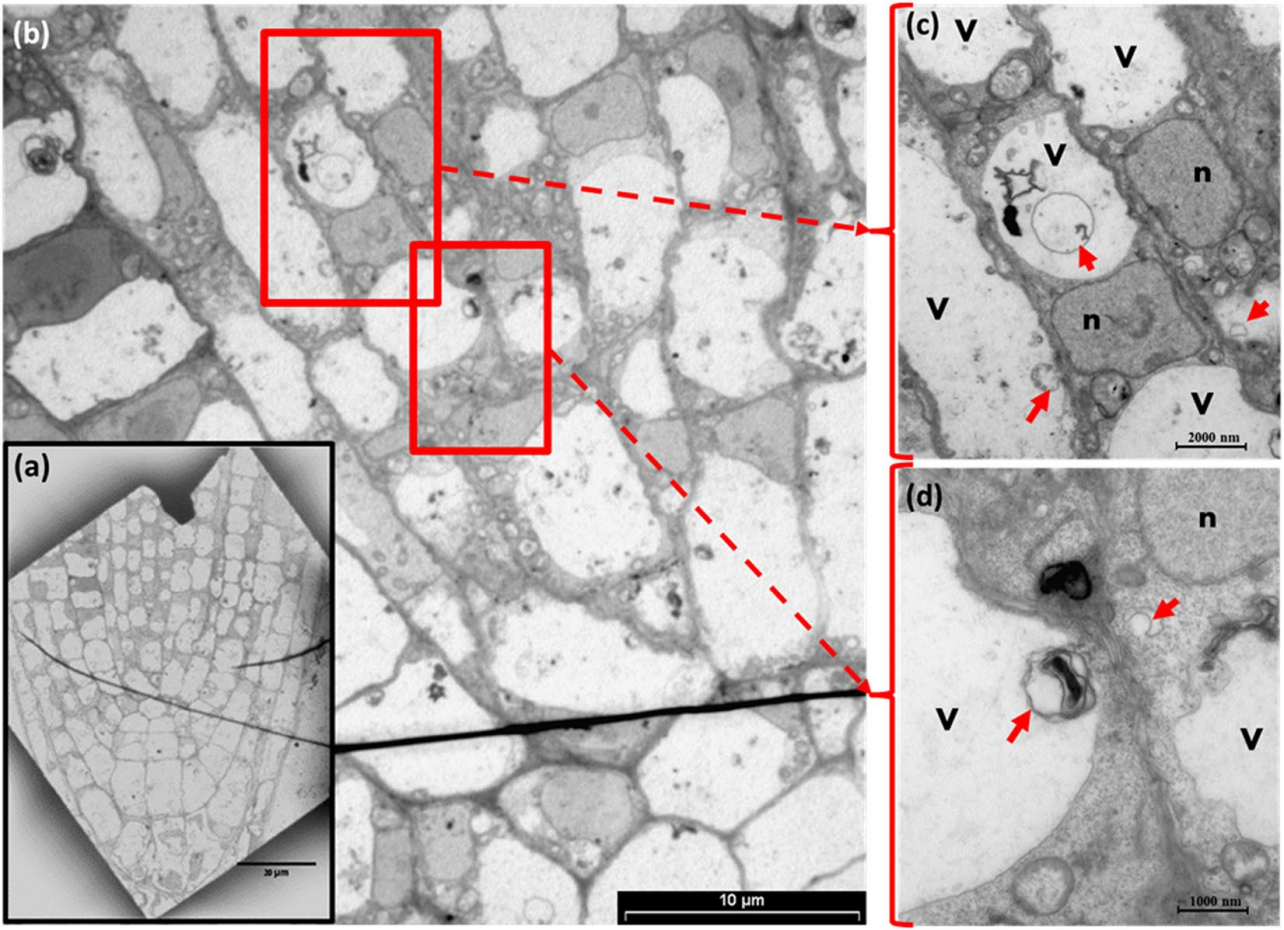
Ultrastructure Analysis of PR cells during lethal salinity. Arabidopsis plants were salt stressed (NaCl, 200 mM). (**a**) Representative TEM micrograph of stressed PR. (**b**) Is a zoom-in micrograph to the meristem core zone presented in (**a**), in which the presence of autophagosome like bodies can be visualized in the vacuoles and cytosol of these cells. (**c,d**) are the magnified regions of highlighted red boxes in (**b**) showing vacuolar and cytoplasmic localized membrane bounded autophagosome like structures. The letters ‘v’ and ‘n’ in (**c**,**d**) indicate the vacuole and nucleus respectively. Red arrows points to autophagosome like structures. Provided are images reflecting two biological replicates.

The presence of autophagosomes in the PR during lethal salinity was further confirmed by TEM analysis, which provided higher resolution micrographs of cytoplasmic as well as vacuole localized phagosomes (Fig. 6a–d) in all the examined cells of the PR meristematic zone. In addition, clear nucleoli and intact nucleus membrane, were observed in all the examined cells of that position (Fig. 6b–d).

### ROS production in PR and LR during lethal salt stress

**Figure 7:**
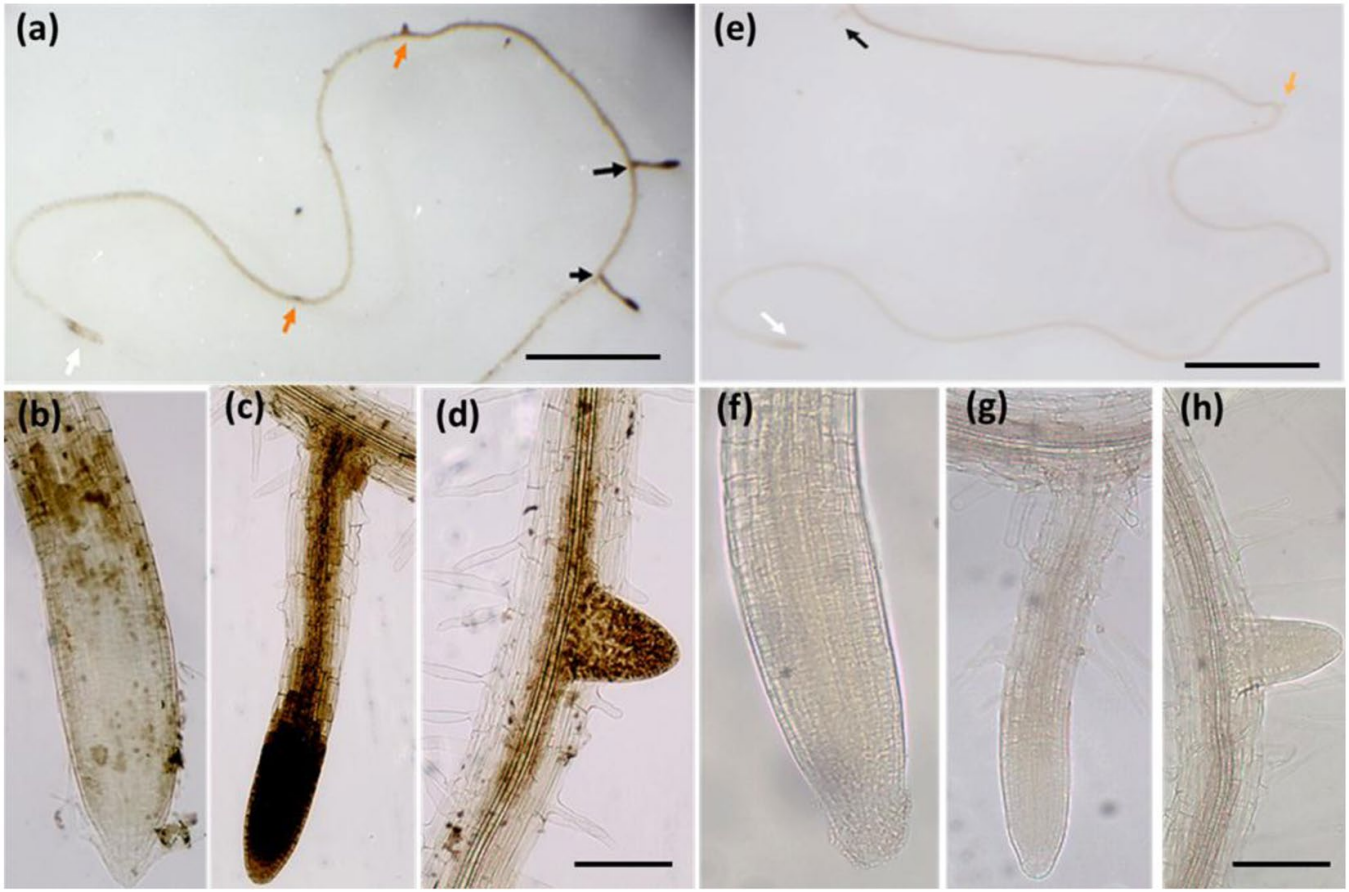
Total ROS in PR and LR by during lethal salinity. Arabidopsis plants subjected to salt stress (200 mM NaCl) at different time points (0 h,12 h,24 h,48 h, & 72 h) were stained with 3,3′-Diaminobenzidine-HCl (DAB-HCl). Images (**a**–**d**) show representative DAB staining in the root 48 HAS. Images (**e**–**h**) present DAB staining in roots of plants that were treated with NaCl for 48 h NaCl in presence of 10 µM DPI. The white arrow indicates PR of (**b**) 48 h salt and (**f**) 48 h salt + DPI whereas (**c** & **g**) black and (**d**,**h**) orange arrows mark the lateral and emerging lateral roots under above mentioned conditions, respectively. (**a**,**e**) Were captured by trinocular Stereo Microscope SZ61 and (b-d &f-g) by inverted microscope AE2000. In (**a**,**e**) scale bar = 0.5 cm. In (**b**–**d**) and (**f**–**h**) the scale bar = 50 µm.

We further studied the presence of ROS in the PR and LR positions at 48 HAS by the H_2_O_2_ indicator DAB and found that the observed ROS distributed differently in both positions: strongly in the LR and very faintly in the periphery of the PR meristem but not inside it. (Fig. 7a–d). The same pattern of uneven ROS distribution in the PR and LR positions during prolonged exposure to lethal salinity was also observed by H_2_DCFDA staining which was used as an alternative ROS indicator (Fig. SI12). Since dead cells do not produce ROS, the lack of the ROS signal from the PR meristem at 48 HAS seems to reflect the high mortality rates observed during that time in the PR position (Figs. 1 and 2). We further treated the salt stressed plants with the NADPH Oxidase inhibitor Diphenyleneiodonium (DPI) and found that the DAB staining signal was completely eliminated from the LR and the periphery cells of the PR meristem (Fig. 7e–h).

To test how DPI affected LR cell viability during prolonged lethal salt treatments, the vital stain PI was applied as mentioned above to SCR::GFP seedlings that were salt stressed for 48 hours in presence of DPI. Confocal microscopy examination of the fluorescent signals in LR of the DPI treated plants detected the PI signal in the cell’s nuclei and failed in detecting any GFP signal, therefore indicating that increased cell mortality has occurred in that root position (Fig. 2c,e). Moreover, increased cell mortality by DPI was also observed in the meristem periphery of the PR, in a region that initially produced positive FDA and DAB signals (see Figs. 1a, 7b).

## Discussion

In this study we measured in Arabidopsis roots the cell’s viability in the PR and LR positions during lethal salinity conditions (200 mM NaCl). By using several vital staining assays, we found that emerging and young LR survived longer than the PR in the *lethal* salinity conditions (Figs. 1–3). To our knowledge, this is the first report demonstrating that phenomena in Arabidopsis roots that were challenged with prolonged toxic salt concentrations.

Our results were further supported by the lack of cell death markers TUNEL and MDC in the young LR during 24–48 HAS. These results indicate that no active PCD process took place at that position during exposure to highly toxic salinity. While the TUNEL positive control showed successful labelling of PR nuclei (Fig. 4a,b), TUNEL positive nucleus labelling was not observed during the high salinity stress in either the PR or the young LR. The lack of TUNEL labelling indicated that no DNA nicking occurred in the salt stressed PR cells, and there is thus no evidence that active AL-PCD took place at either position. On the other hand, strong induction of MDC labeled authophagosomes was detected in the PR (and not in the LR) 24 HAS (Fig. 5a–c). The presence of these autophagosomes in that position during salt stress was further confirmed by TEM analysis to be localized in both the cytoplasm as well as the vacuole (Fig. 6). Considering the high mortality rates observed in the PR during this stress duration (40–80%, Fig. 1), the presence of these phagosomes is indicative of an active A-PCD process that takes place in that position during lethal salinity. Moreover, AL-PCD is characterized by sever nuclei shape distortion and disintegration^40^, though TEM analysis revealed that the nuclei in all of the PR examined cells had normal appearance with intact nucleus membranes (Fig. 6). Unlike necrosis, in tissues undergoing PCD, cell death is not completely synchronized. Therefore, a few cells are expected to die late as was observed in our viability staining assays in which during 48 HAS 4–7 percent of the PR peripheral cells still exhibited positive FDA signal (Fig. 1a).

Thus, these observations provide another line of evidence that AL-PCD does not take place in the Arabidopsis PR during lethal salinity, but rather A-PCD. Since lethal salinity was shown to induce positive TUNEL labeling in nuclei of PR cells in tomato^41^ and rice^42^, we suggest that lethal salinity can induce different types of PCD in the PR of different species.

Our results also suggest that cell death is differentially regulated in each of the two root positions during lethal salinity. A key player that might be involved in such regulation is NOX induced ROS (known also as RBOH, or N ADPH Ox idase) that is well known to be involved in Autophagy induction^43^. In this study we further found that during lethal salinity NADPH-Oxidase activated ROS accumulated to high levels in the LR but not in the PR (Fig. 7a), in an opposite manner to the autophagosome presence in these positions (Fig. 5). Therefore, this ROS may be unrelated to the A-PCD induction and is in line with reports of NOX-independent Autophagy induction^44^. Nevertheless, blocking this ROS by DPI increased cell mortality in the young LR during lethal salinity (Figs. 2c,e), which indicates that NADPH Oxidase activated ROS is required for the young LR cell survival in *lethal* salinity conditions, and correlates with reports describing the essential roles that ROS plays in plant response to abiotic stresses^45,46^.

In this study we also found that the PR meristem acquired salt tolerance as the meristem aged and differentiated. Young dividing meristematic cells of 2DAG plants were highly sensitive to salt stress, but at age of 8DAG exhibited higher survival rates (Fig. 3). Indicating the phenomenon of age acquired salt tolerance (AAST). This finding is in line with Lutts *et al*.^47^ which reported that young rice seedlings were most sensitive to salt during the vegetative stage. Nevertheless, an opposite trend was observed in the LR, where the young meristems which were highly salt tolerant (much more than the 2DAG and 8DAG PR meristems), lost completely their tolerance as the LR elongated beyond 400 µm and the meristem became older and more differentiated (Fig. 3).

Interestingly, during 48 HAS the ROS production in elongated LR’s was higher than young-emerging lateral primordia (Fig. 7c,d). Since the elongated LR’s (which were shorter than 400 µm), exhibited higher death rates at 48 HAS as compared with the young-emerging LR’s (shorter than 100 µm) (Fig. 1c. 21.3% and 5.2%, respectively), their excessive ROS production may have crossed a certain threshold which can induce death, and is in line with reports connecting ROS with cell mortality^46^. Thus, this excessive ROS may play a role in the young LR reduced salt resistance during elongation.

The improved salt tolerance of the young LR reported here can be explained on the basis of surface area salt exposures: the young-short LR have less surface area which is exposed to salt as compared with the elongated LR which due to their increased surface area, suffers from increased salt exposure. Consequently, as the LR elongates, the increased exposure to salt accelerates death rates, which beyond 400 µm length reaches the same high death rates of the PR. Nevertheless, at the cellular level, we have also shown here that even young LR cells are significantly plasmolysed by the salt (Figure SI11), thus indicating that these cells are actually not protected from the salt. Therefore, further work (such as cellular NaCl quantifications and NaCl sequestration activity), is needed to clarify whether young-short LR cells experience lower degrees of salt stress than elongated LR cells.

In a broader perspective, since the major movement of salt in soils occurs through the medium of water, water irrigation practices have been shown to affect soil salinity and form various types of salt gradients^48^. Moreover, in semi-arid/arid regions, salts tend to remain and accumulate closer to the soil surface, whereas in rainy regions, salts are constantly washed from the surface deep into the ground, thus creating salt increase with depth^49,50^. Therefore, in the latter regions, avoiding downward growth by eliminating the PR meristem while maintaining sideway growth ability by preserving the LR viability may be indicative of a salt escape mechanism that can increase the plant fitness to that environment.

In summary, we have shown here that, in Arabidopsis, emerging and young LR survives lethal salinity longer than the PR. In addition, age acquired salt tolerance (AAST) was observed in the PR but not in the LR. The mortality of the PR cells was attributed to induction of A-PCD, while no signs of active PCD processes were observed in the LR cells, which required NADPH-Oxidase activated ROS for their survival. Resolving the transcriptome of the different LR cell types during lethal salinity, which is beyond the scope of this study, holds the potential to reveal novel salt stress coping mechanisms and provide new tools to face the ongoing challenges of soil salinization^1,2^.

## Methods

### Plant material and growth

The following lines of Arabidopsis thaliana plants were used in this study: Wild type (wt, ecotype Columbia-0),SCR::GFP, WOL::GFP, COR::GFP^11^ and WOX5::GFP^51^. Sterilized seeds were planted on 0.8% agar plates (Caisson lab, USA) supplemented with ½ MS Basal Salt medium (Caisson lab, USA) under white light (120–130 µmol/m^2^ sec) in a growth room with day/night cycles of 16 h at 24 °C and 8 h at 18 °C, with humidity of 40–60%. For salt stress, the 7 day old seedlings were transferred to liquid ½ MS media supplemented with 200 mM NaCl.

### Viability assays

The vital stains Fluorescein diacetate (FDA, Sigma) and Propidium Iodide (PI, Sigma) were applied simultaneously as reported by^52^. Cell viability was also determined in SCR::GFP plants as reported by^53^. The fluorescent signals were detected by laser scanning confocal microscope - LSM710 (Zeiss) with a Plan-Apochromat 20 ×0.8 numerical aperture air objective lens. FDA and GFP Ex/Em was 488-nm and 525-nm, PI Ex/Em was 555-nm and 580-nm. The transmitted light (bright-field) images were recorded for each image. Images were scanned using the same conditions of the pinhole, gain, laser power (2%), and detector offset in each experiment.

### TUNEL stain

TUNEL staining was performed as previously described^54^. The shoots were removed and nick-end labeling was carried out on whole root using a “Dead End Fluorometric TUNEL kit” (Promega G3250) according to the manufacturer’s protocol. To afford a TUNEL positive control, the fixed and permeabilized non-stressed plants were incubated in 10 units/ml DNase I (Progema Cat. # M6101) for 20 minutes. The samples were observed by confocal microscopy LSM700 (Zeiss). 488 nm laser lines was used to capture images of fluorescein-12-dUTP labelled TUNEL positive nuclei -and 561 nm laser line for PI labelled nuclei.

### Monodansylcadaverine (MDC) staining

MDC (Sigma) staining was performed as described previously^39^. In brief, plants were washed with DDW and dipped in sufficient volume of 0.05 mM MDC in PBS for 30 min. Fluorescence was captured using EVOS M5000 fluorescence microscopy using DAPI-specific LED cube (357/44 nm Excitation; 447/60 nm Emission) fitted with a high-resolution CMOS camera.

### TEM Ultrastructural studies

The presence of Autophagosome in the PR was also observed by TEM. The PR zone of stressed Arabidopsis plants were excised under trinocular stereo microscope (SZ61- OLYMPUS) using sharp razor blade and fixed in 5% Glutaraldeyde in 0.1 M Cacodylate buffer (pH 7.4) 3 hours at room and transferred to 4 °C for continuation of fixation overnight. The samples were further processed according to Galsurker *et al*.^55^. The final sections were placed on grids and sequentially stained with uranyl acetate and Lead citrate for 10 minutes each and viewed with Tecnai 12 TEM 100 kV (Phillips, Eindhoven, the Netherlands) equipped with MegaView II CCD camera and Analysis version −3.0 software (SoftImaging System GmbH, Münstar, Germany).

### Reactive oxygen species (ROS) detection

H_2_O_2_ was detected by 3,3′-diaminobenzidine (DAB) staining, as previously described^56^. Salt stressed Seedlings with and without 20 μm diphenyleneiodonium (DPI)^13^ were treated with DAB Staining buffer for 60 min and the reaction was stopped by adding Ethanol: glycerol: acetic acid (3:1:1). Images were captured using trinocular and inverted microscopes mentioned above. H_2_O_2_ was also detected by 2′,7′- dichlorodihydrofluorescein diacetate (DCFDA) as in^57^. Salt treated plants were washed and treated with 50-μM (DCFDA) in water. After 30 min of incubation, the ROS-specific fluorescence was detected using a confocal microscope LSM710 (Zeiss) using Ex/Em 488/525 nm.

### Statistical and fluorescence analysis of images

Image analysis was performed using Zen Blue software (Zeiss) and ImageJ (https://imagej.nih.gov/ij/). All the statistical analysis was carried out using Graphpad Prism (https://www.graphpad.com/scientific-software/prism/).

## Supplementary information

Supplementary Information.
