# Supplementary material for: Laterals take it better – Emerging and young lateral roots survive lethal salinity longer than the primary root in Arabidopsis
Source: Sci Rep. 2020 Feb 24;10:3291. doi: 10.1038/s41598-020-60163-7 (PMC7040039; doi:10.1038/s41598-020-60163-7)
Supplement: Supplementary file 1 — Supplementary Information. [file 41598_2020_60163_MOESM1_ESM.pdf]

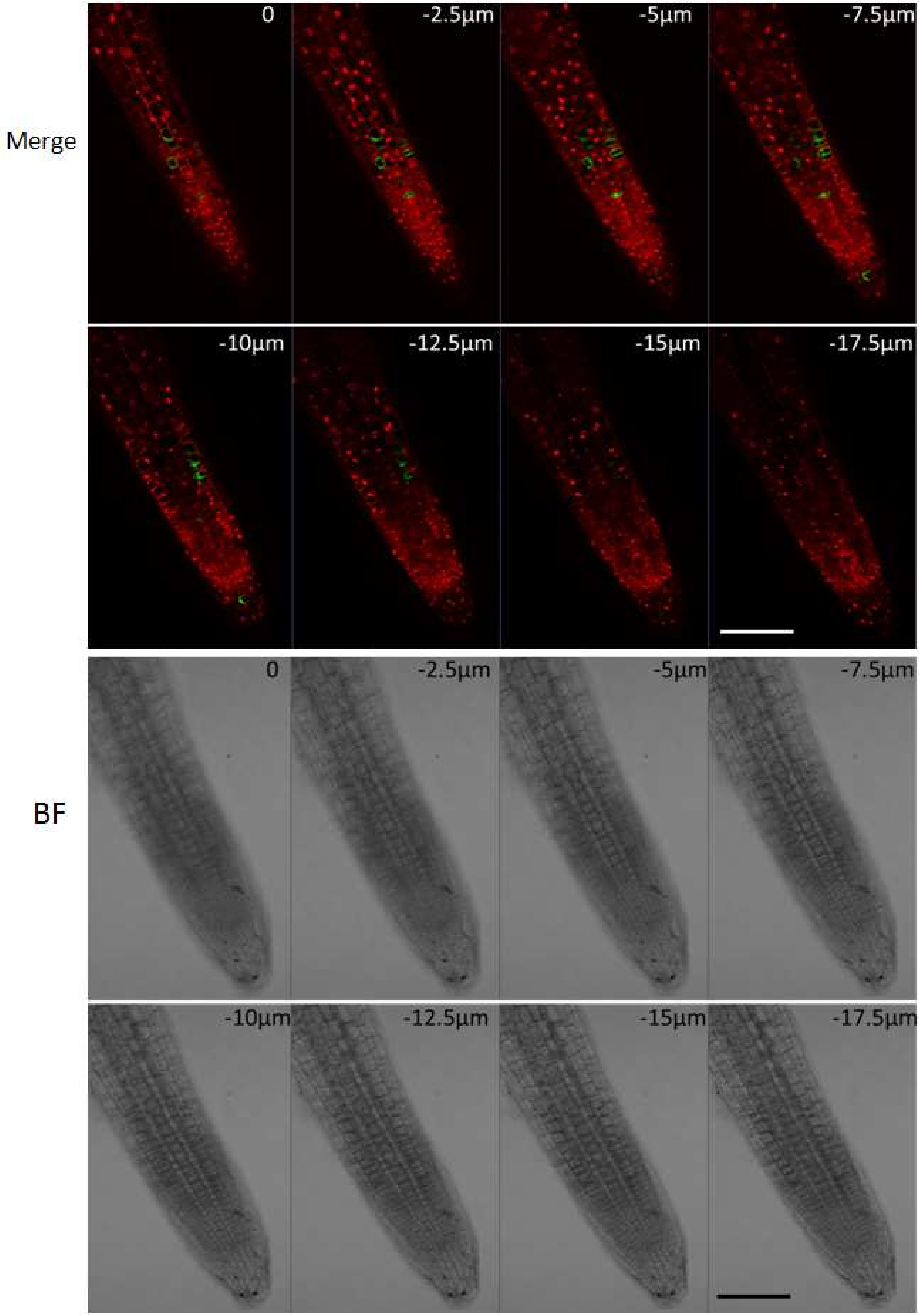

Figure SI1 –Complete Z-stack images of Fig 1a. Shown are the merge of FDA and PI fluorescence and bright field images. The numbers at the top-right corner of each image indicate its depth along the Z axis. Scale bar =100µm.

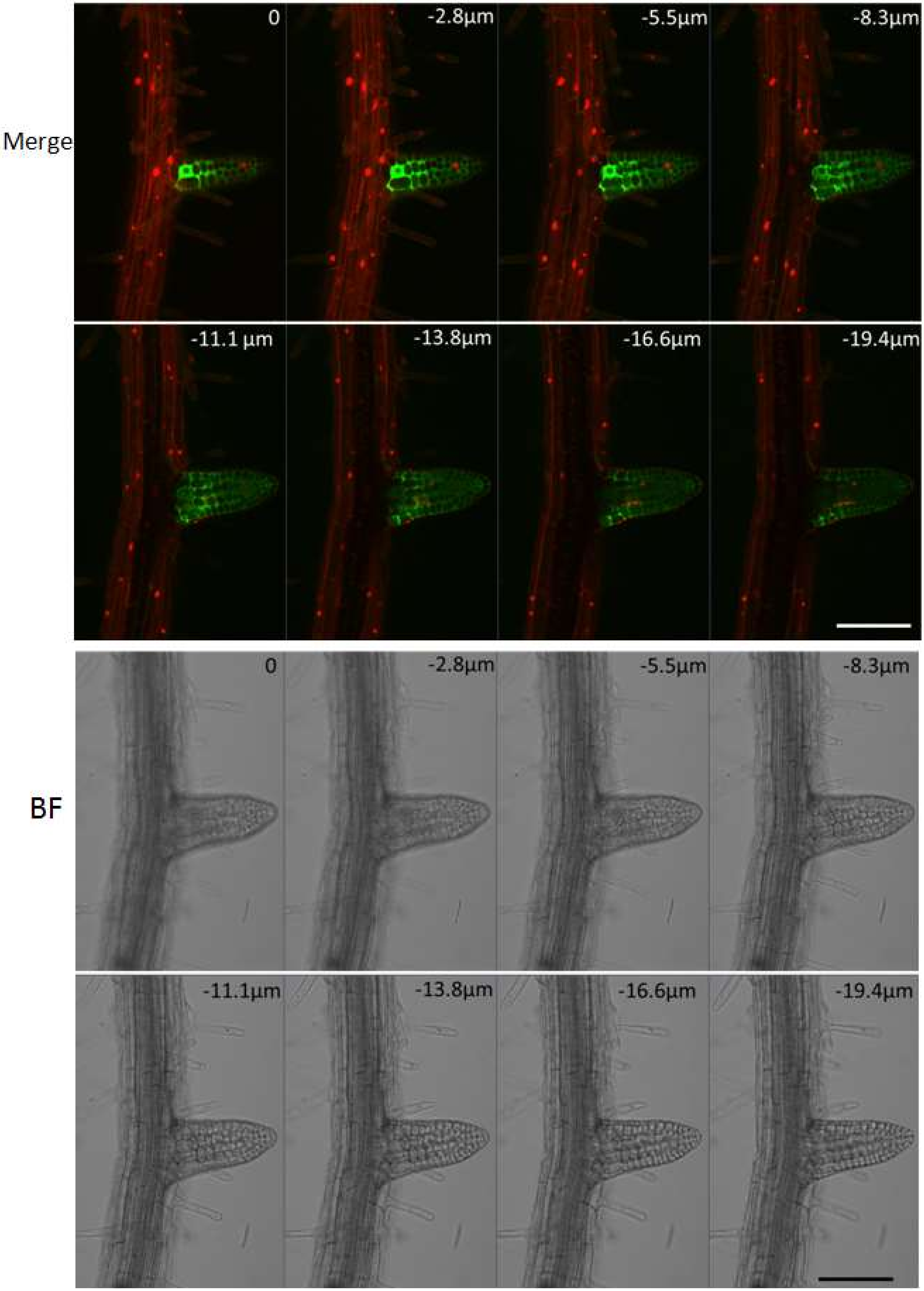

Figure SI2 –Complete Z-stack Images of Fig 1b. Shown are the merge of FDA and PI fluorescence and bright field images. The numbers at the top-right corner of each image indicate its depth along the Z axis. Scale bar =100µm.

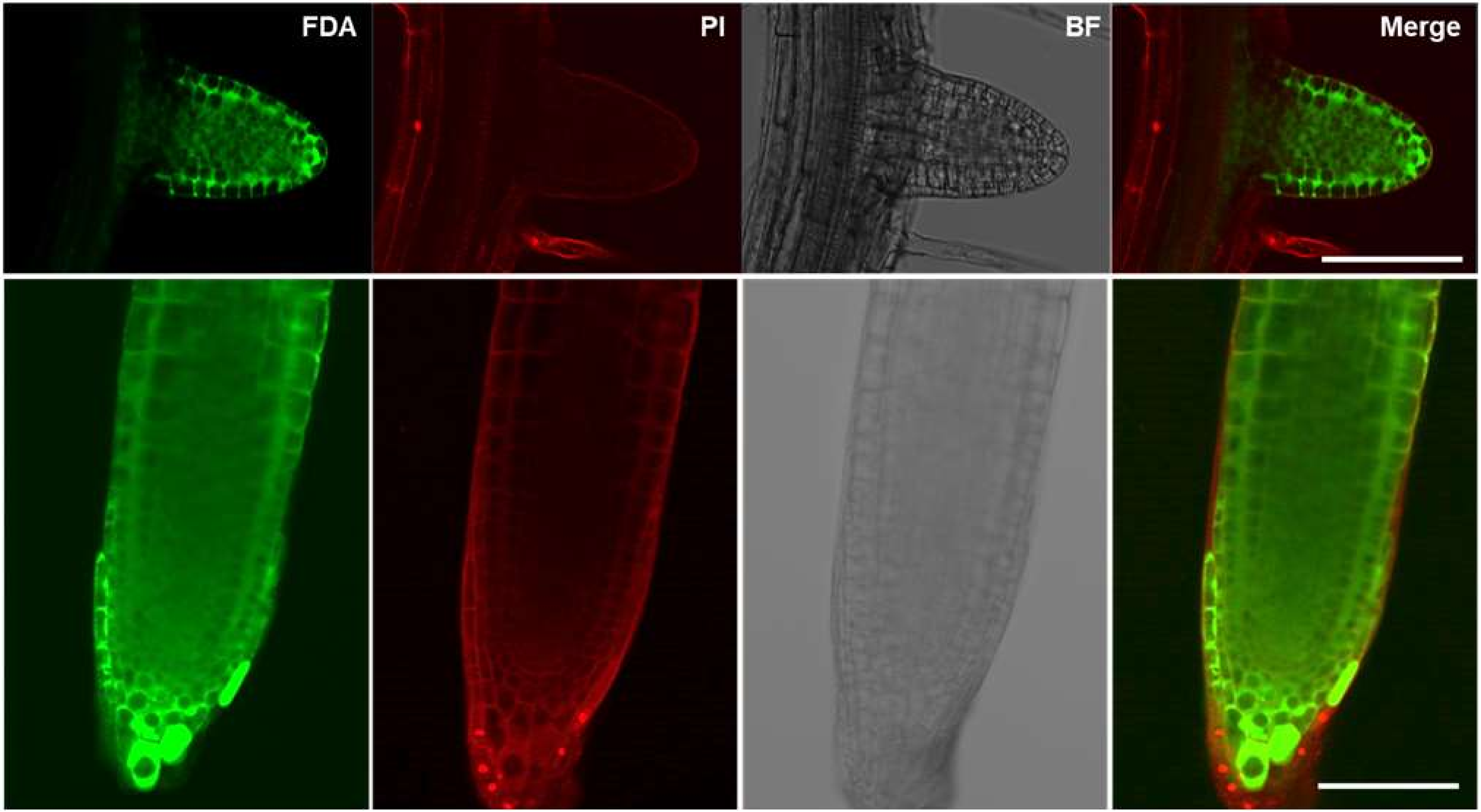

Figure SI-3 –Confocal images of FDA+PI in the PR and LR during control conditions.
Confocal microscope images of FDA and PI fluorescence in the LR (upper panel) and the PR (lower panel) of 9 days ½ MS grown control Arabidopsis seedlings. Scale bar = 100μm. (Note that apart of few root cap cells in the PR, the PI stained the plasma membrane but not the nucleus).

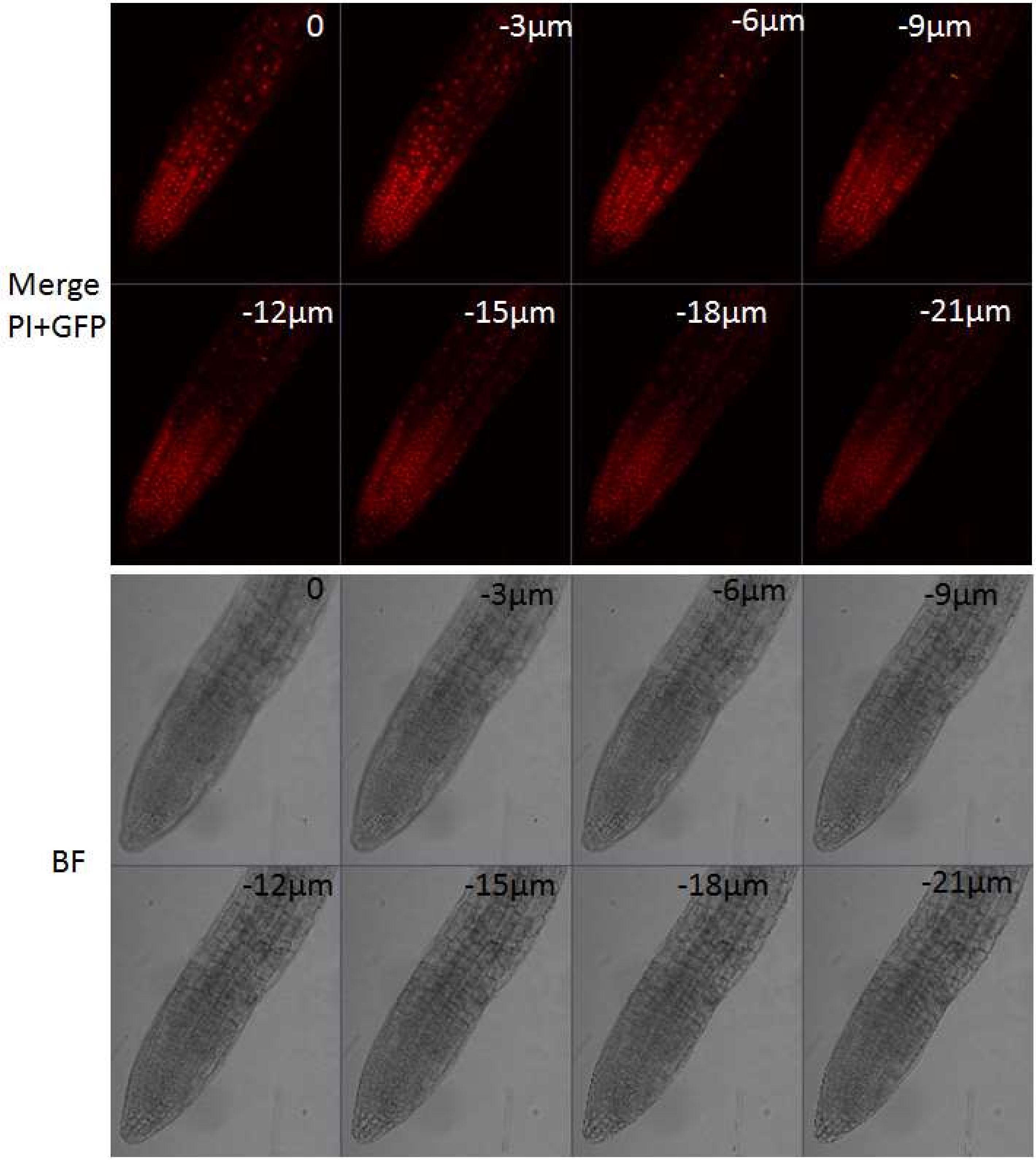

Figure SI-4– The complete Z-stack Images of Fig 1e . Shown are the merge of GFP and PI and bright field images. The numbers at the top-right corner of each image indicate its depth along the Z axis.

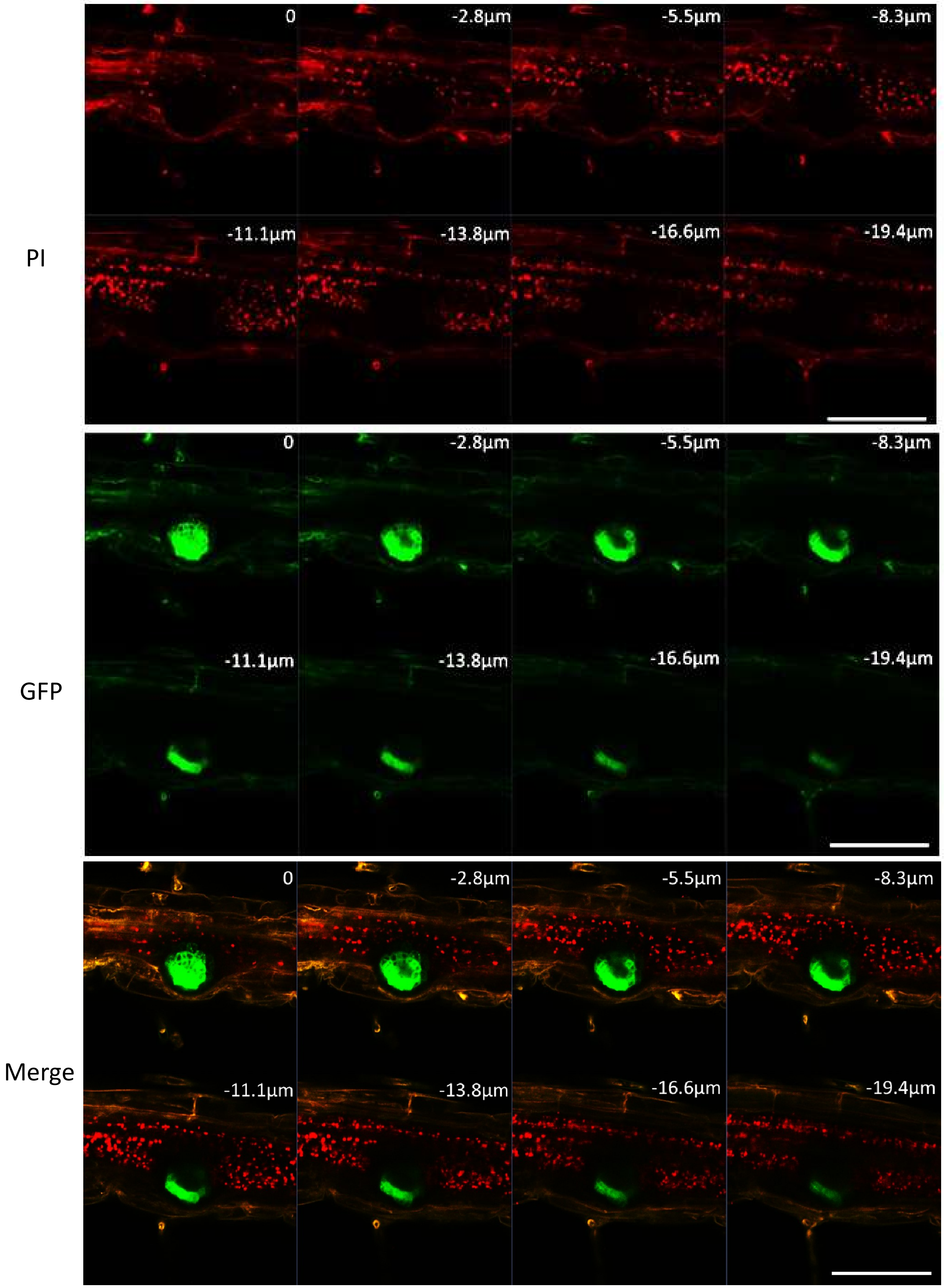

Figure SI5– The complete Z-stack Images of Fig 1f. Shown are the images from the separate PI and GFP filters as well as their merged (PI+GFP) images. The numbers at the top-right corner of each image indicate its depth along the Z axis. Scale bar = 100µm.

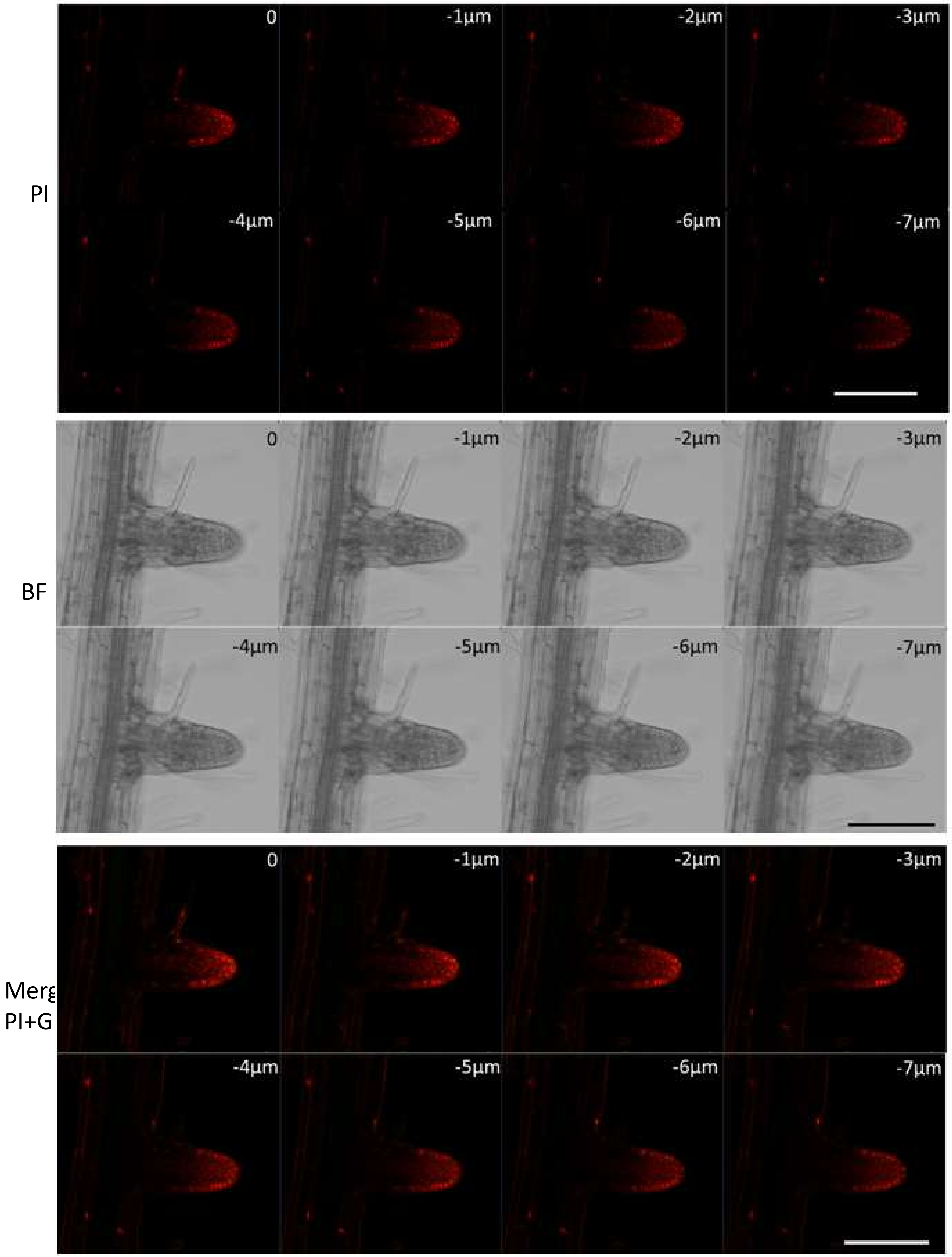

Figure SI6– The complete Z-stack Images of Fig 1g . Shown are the PI, bright-field and merge (GFP and PI) images. The numbers at the top-right corner of each image indicate its depth along the Z axis. Scale bar = 100µm.

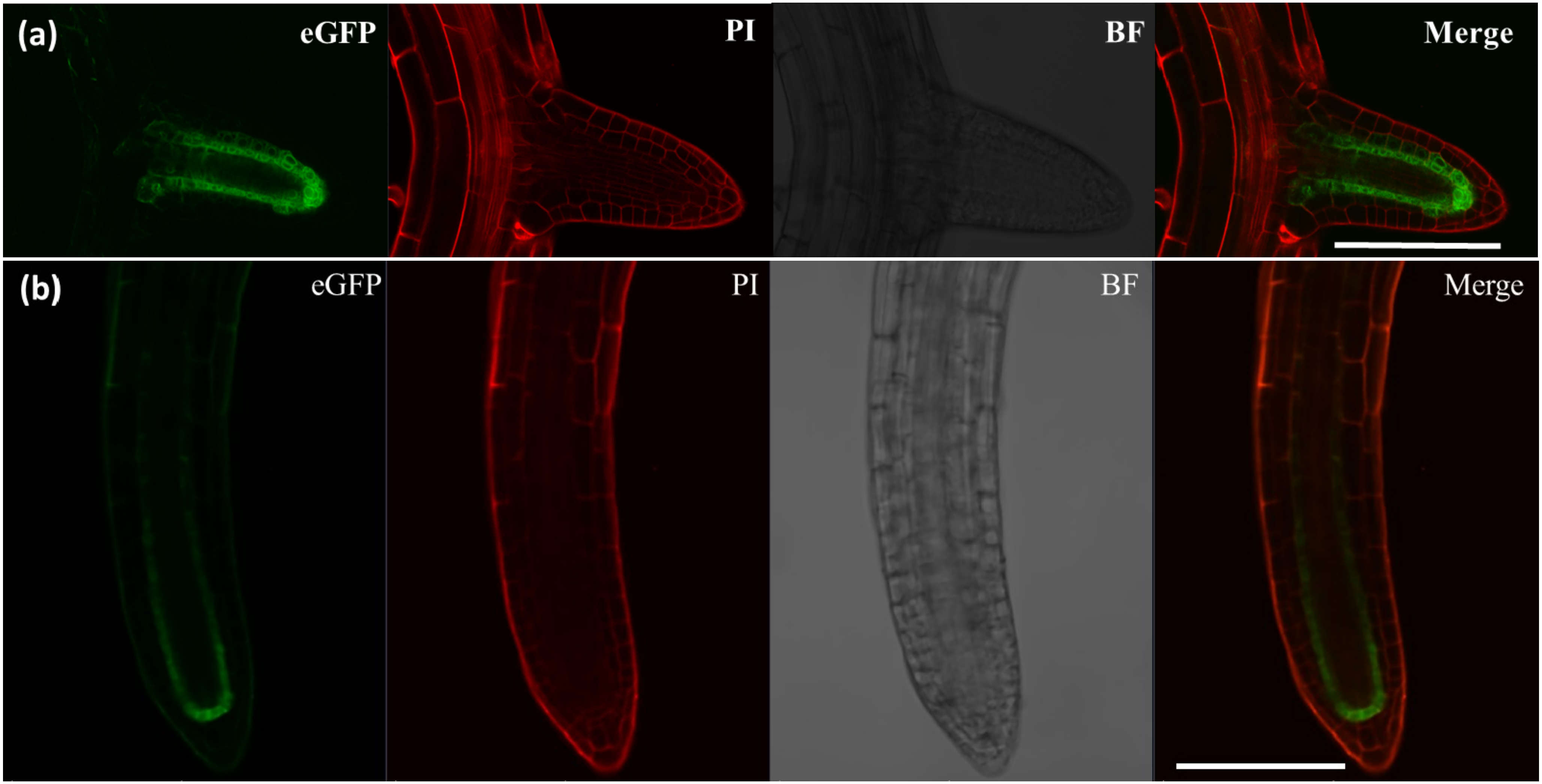

Figure SI-7. Confocal images of LR and PR of SCR::GFP stained with PI of control Maximum intensity confocal images of lateral root (a) and Primary root (b) of Scarecrow::GFP stained with PI under control condition. Scale bar=100 µm.

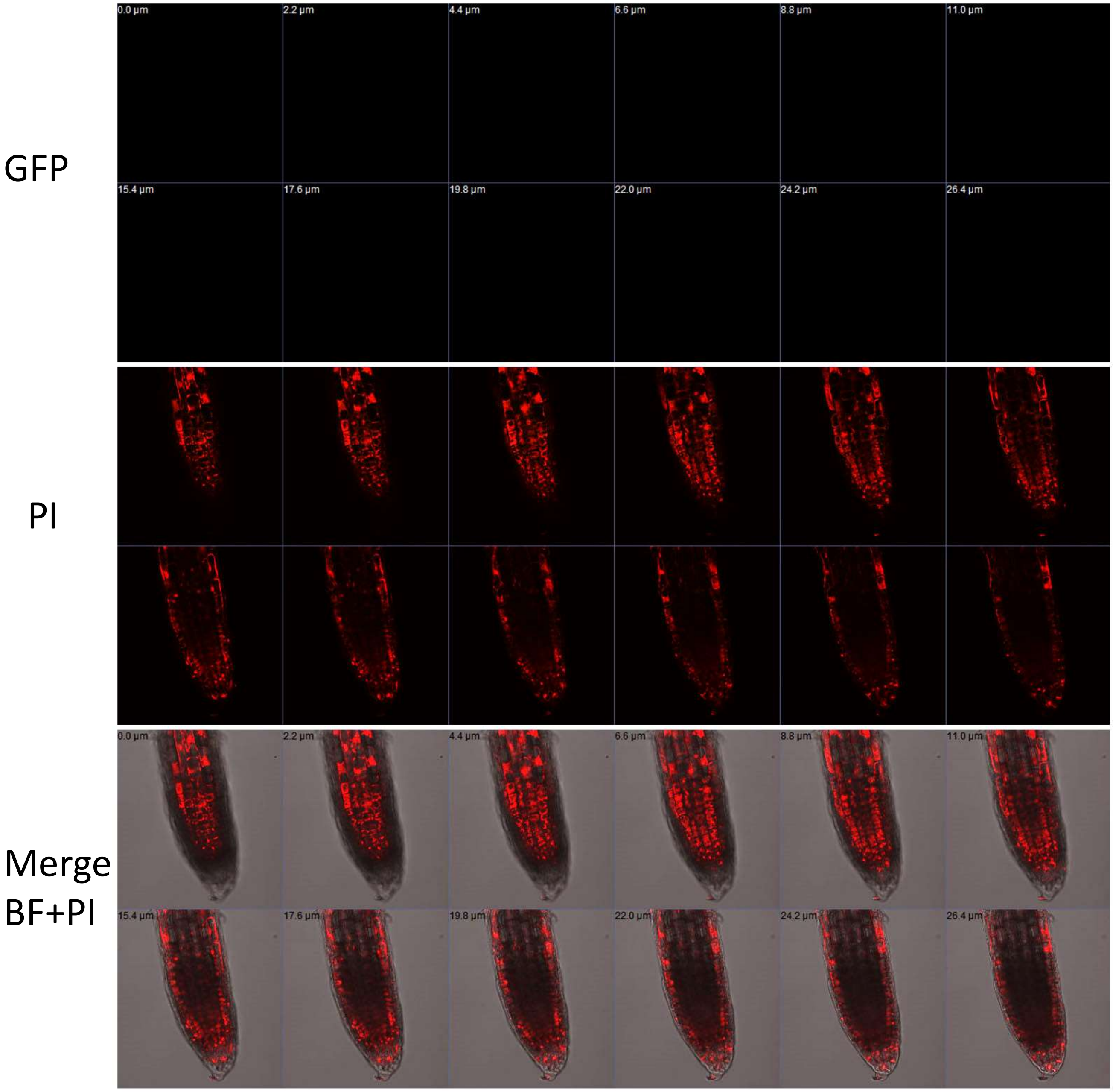

Figure SI8 –Complete Z-stack Images of Fig 3a. Shown are the merge of GPF,PI and Merge (PI+BF)fluorescence and bright field images. The numbers at the top-right corner of each image indicate its depth along the Z axis.

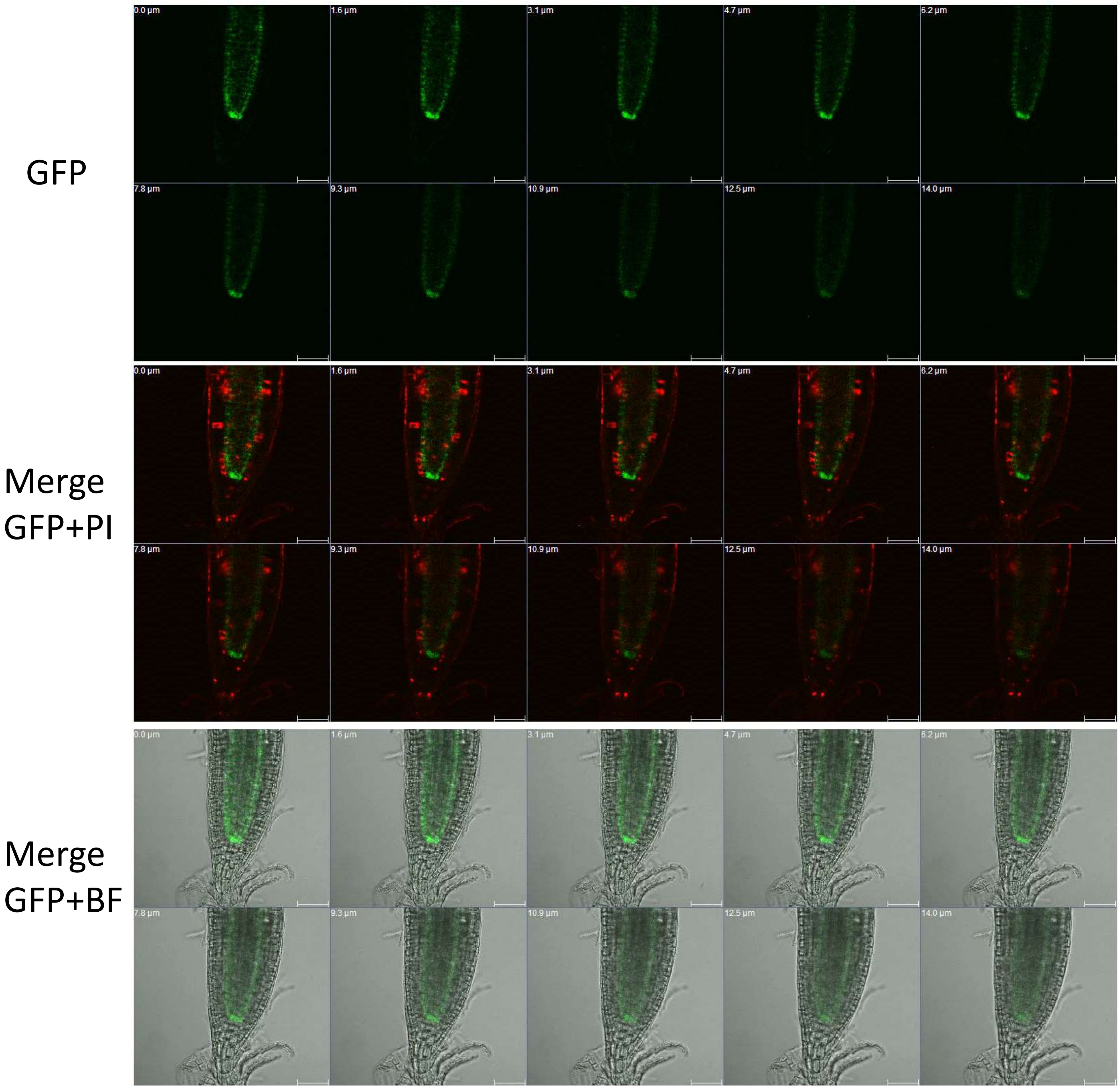

Figure SI9 –Complete Z-stack Images of Fig 3b. Shown are the merge of PI ,GPF and Merge (GFP+PI and PI+ BF) fluorescence and bright field images. The numbers at the top-right corner of each image indicate its depth along the Z axis. Scale bar = 50µm

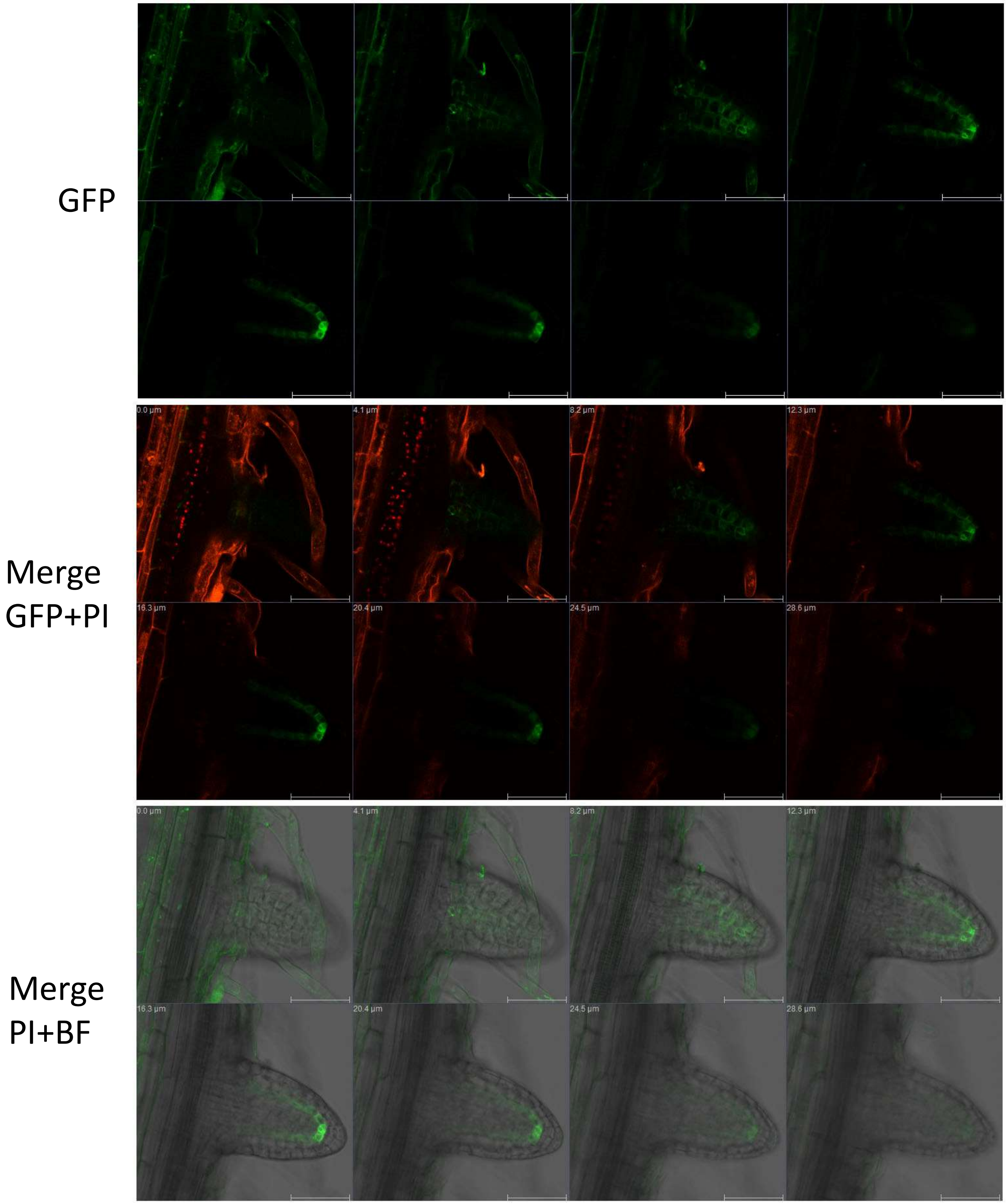

Figure SI10 –Complete Z-stack Images of Fig 3c. Shown are the merge of PI ,GPF and Merge (GFP+PI and PI+ BF) fluorescence and bright field images. The numbers at the top-right corner of each image indicate its depth along the Z axis. Scale bar = 50µm

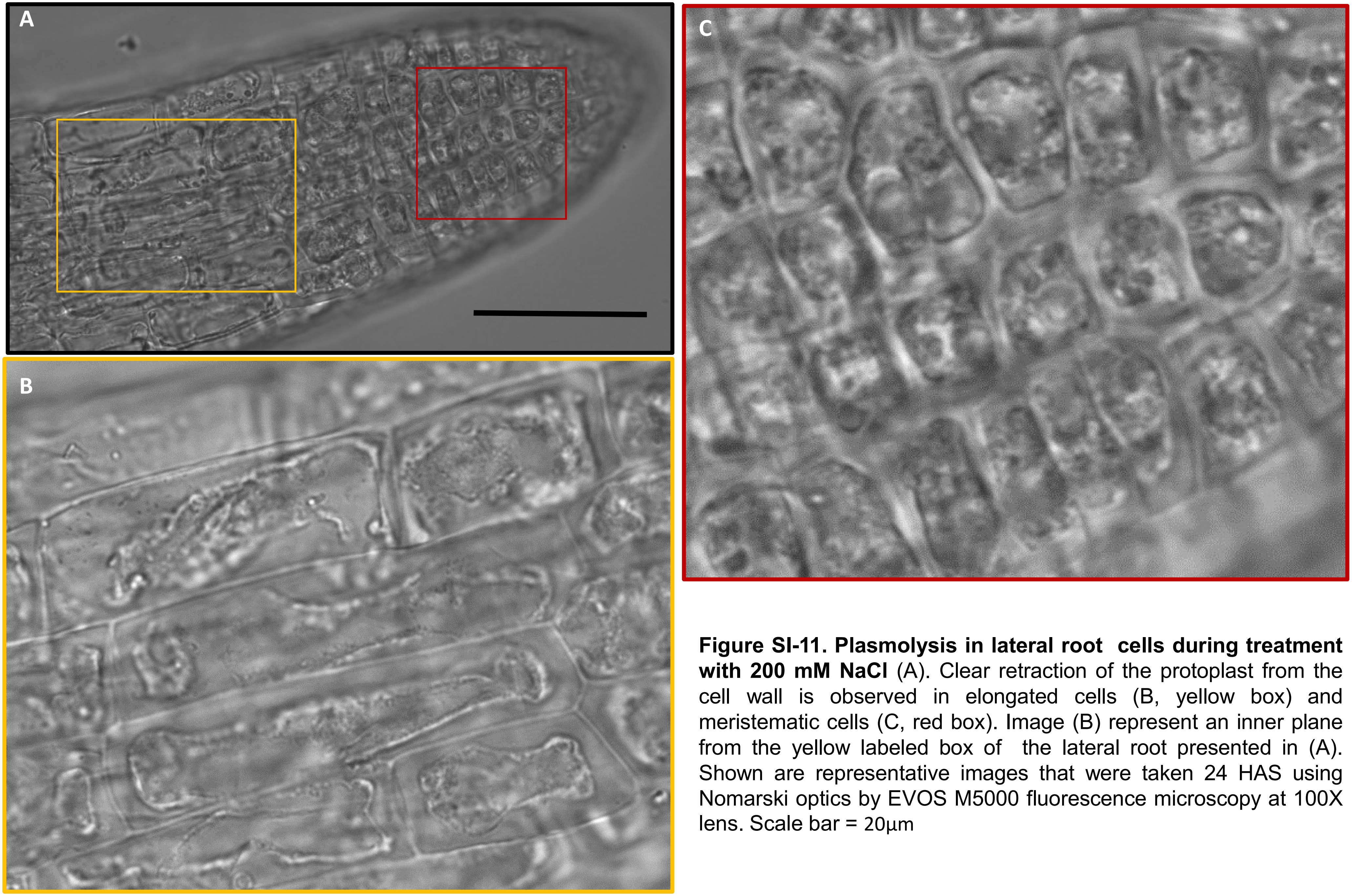

Figure SI-11. Plasmolysis in lateral root cells during treatment with 200 mM NaCl (A). Clear retraction of the protoplast from the cell wall is observed in elongated cells (B, yellow box) and meristematic cells (C, red box). Image (B) represent an inner plane from the yellow labeled box of the lateral root presented in (A). Shown are representative images that were taken 24 HAS using Nomarski optics by EVOS M5000 fluorescence microscopy at 100X lens. Scale bar = 20µm

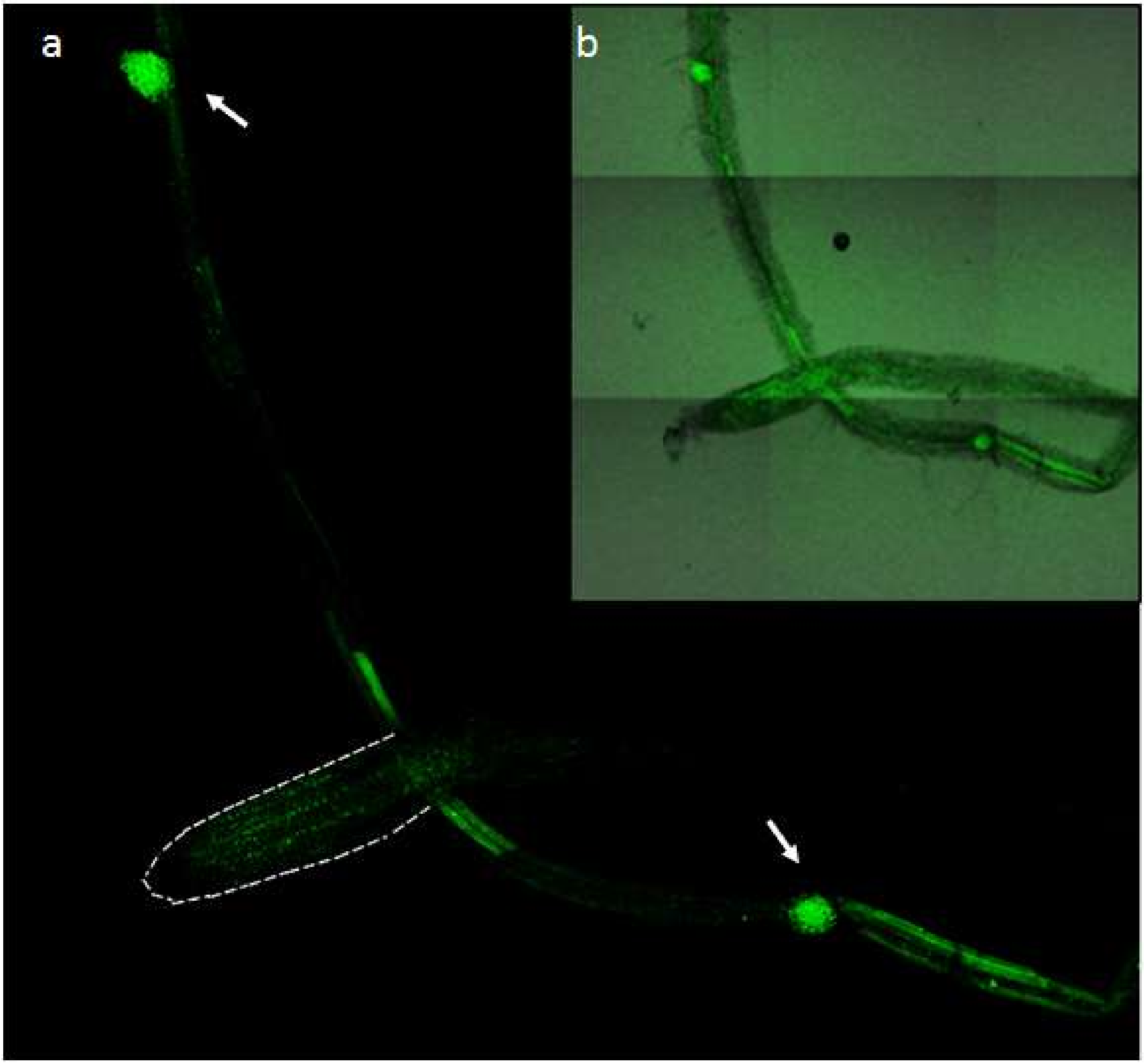

Figure SI-12. ROS detection in PR and LR during lethal salinity using DCF-DA.
Confocal images of DCF-DA fluorescence in roots of Arabidopsis plants treated for 24 hours with 200mM NaCl. White arrow in (a) points to emerging lateral roots while the broken white line marks the primary root. Inset (b) is the merge of the bright field and the green fluorescent channel. DCF-DA incubation and detection was performed as mentioned in methods.

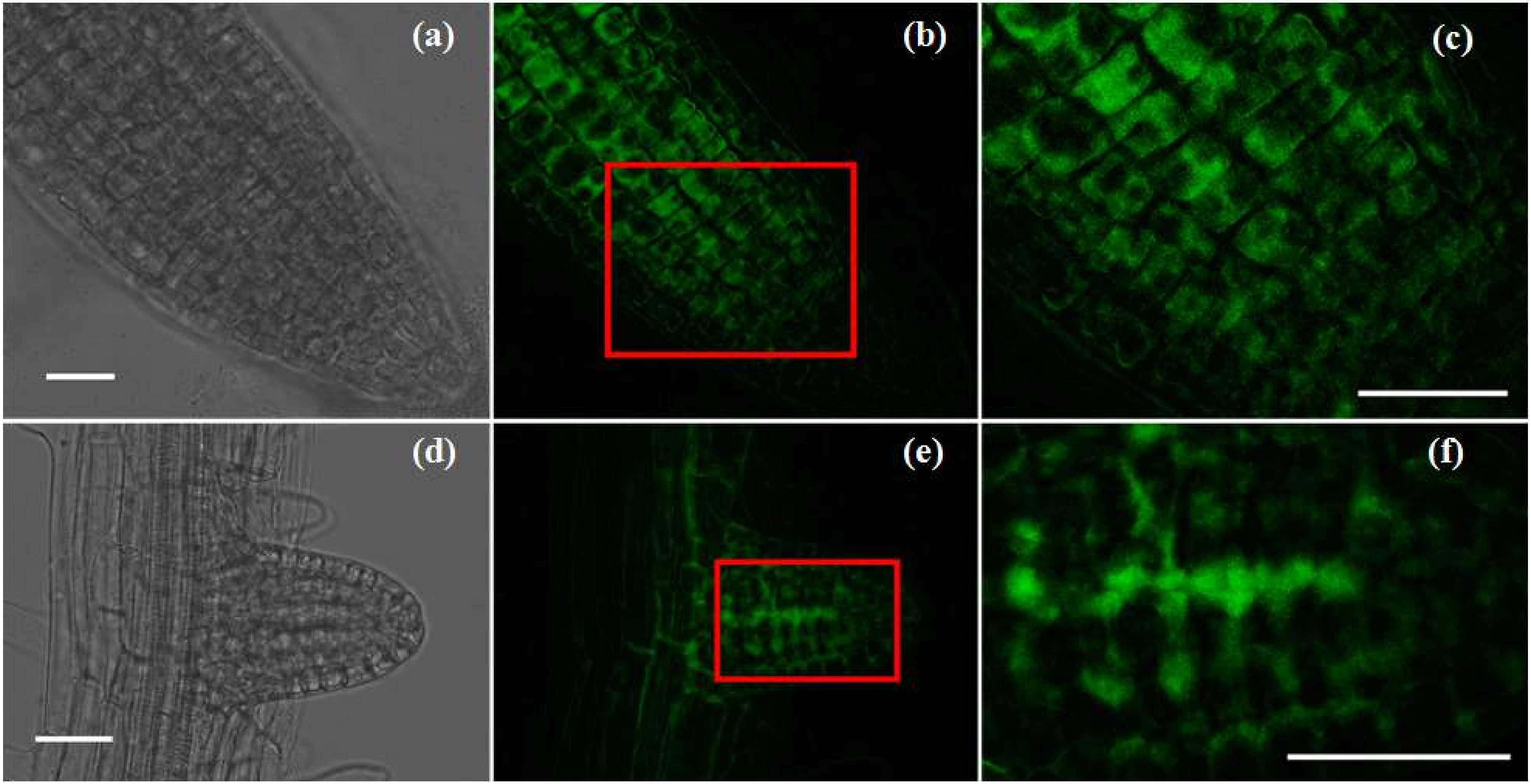

Figure SI 13 -Monodansylcadaverine (MDC)-Staining in primary and lateral root of control plants. Arabidopsis seedlings grown under control condition were stained with MDC as in figure 4, then analyzed by EVOS M5000 fluorescence microscopy using DAPI-specific LED cube. No MCD labelled bodies were observed either in the PR (a-c) or the LR (d-f). Scale bar = 20μm.
